# Does the Australasian “Health Star Rating” Front of Pack Nutritional Label System Work?

**DOI:** 10.3390/nu8060327

**Published:** 2016-06-01

**Authors:** Robert Hamlin, Lisa McNeill

**Affiliations:** Department of Marketing, University of Otago, PO Box 56, Dunedin 9001, New Zealand; lisa.mcneill@otago.ac.nz

**Keywords:** front of pack, nutrition label, health star rating, traffic light label, FOP, TLL, HSR

## Abstract

This article describes an experiment to measure the impact of the Australasian “Health Star Rating” front of pack nutritional label system on consumer choice behaviour. This system presents a one-half to five star rating of nutritional quality via the front facings of food product packages. While this system has been recently rolled out across Australasia, no test of its impact on food choice has been conducted. A sample of 1200 consumers was recruited on exit from supermarkets in New Zealand. A 2 × 2 factorial design was used with two levels of cold cereal product nutritional status (high, five star/low, two star) and two levels of the Health Star Rating label (present/absent). The dependent variable was revealed choice behaviour. The results indicated that the presence of the label had a significant depressive effect on consumer preference, but that this impact was not moderated in any way by the nutritional status expressed by the label. The result represents a significant functional failure of the Health Star Rating label in this research environment. The nature of the failure is consistent with the consumers processing the label in much the same way as the nominal brand cues that dominate the retail food packaging.

## 1. Introduction

Front of pack (FOP) nutritional labels have attracted considerable attention in the marketing and nutrition research literature over the last two decades [1,2]. The purpose of an FOP nutritional label is to offer guidance to consumers on the nutrition status of the food product to which it is attached. It has been a requirement of most developed countries that food products carry specific information as to their ingredients, source and nutritional content on a dedicated nutrition information panel (NIP). However, this information is usually complex, cryptic and placed on areas other than the front/prime facing, where it is unavailable for use in the fast visual evaluations that characterize the large majority of fast moving consumer goods (FMCG) and food purchases [3].

**Figure 1:**
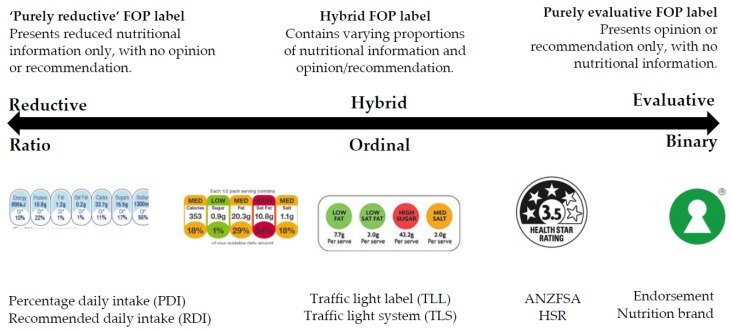
Evaluative/reductive based typology of front of pack (FOP) labels ([6], Reprinted with permission).

FOP labels seek to augment this existing nutritional information, and to present it in a manner that makes it available as an input to these high speed consumer decisions. There is as yet no consensus as to the best way that this might be done, and consequently a very wide variety of third party FOP label formats are proposed or in use around the world [4]. In addition there are an increasing number of “proprietary” FOP labelling systems that are the private property of individual manufacturers and retailers, which further increases communication “noise” and the potential for consumer confusion [5]. Despite this variety, there is one underlying and uniform theoretical “backbone” to all these labels. They can all be placed upon a single continuum anchored at one end by FOP labels that are fully reductive in nature, and at the other by FOP labels that are fully evaluative (Figure 1).

A fully reductive FOP label presents factual information only, without expressing any opinion. The percentage daily intake (PDI) label shown in Figure 1 is an example of this type [7]. A fully evaluative label presents opinion only, without information. The “Swedish Keyhole” label shown in Figure 1 is an example of this type [8]. The keyhole image transmits no factual information whatsoever; it merely expresses an unqualified third party opinion that this product is nutritionally “good”. In between these two extremes lie a variety of hybrid FOP systems that contain elements of both information and opinion [9]. The Traffic Light Label (TLL) is the most widely deployed hybrid FOP label, and a wide range of nutritional information (e.g., percentage daily intake of fat) and opinion (related TLL colour) mixes are available within the broad TLL format (Figure 1).

There is a second typological continuum, in addition to the anchored continuum described above, that is based upon manner in which the information is transmitted. Fully reductive formats, such as the PDI label, are “ratio” in nature. They express information in regular units with a specific zero point. Purely evaluative labels are “binary” in nature; they express the third party opinion, by the presence or absence of the relevant mark (Either the food product has a Swedish keyhole, or it does not—there are only two available states). The intermediate types are all ordinal in nature. They can express several statuses for the product. This ordinal hierarchy can get quite complex. For instance, the four dimension, three status (green amber/red) traffic light mark, that is in the middle of the continuum in Figure 1, can express nine different nutritional “scores” in any one of 3^4^ (81) different ways. If the four traffic lights of a four light array are taken to be fully compensatory, with “red” scoring zero, “amber” scoring one and “green” scoring two, then there the label is able to express nine possible discrete consolidated nutritional “scores” for the product, ranging from four reds (zero) to four greens (eight). The 81 possible combinations are distributed among these nine scores according to a binomial distribution as follows: 0:1:, 1:4, 2:10, 3:16, 4:19, 5:16, 6:10, 7:4, 8:1.

These two continuums, the nature of the information that is transmitted and the manner in which it is transmitted by the FOP, run in parallel with one another, and are represented by the double-headed arrow in Figure 1. Several academic reviews of the extensive body of FOP research exist within the literature [1,2,10,11]. Two recent reviews have observed that the vast majority of the research to date has been developmental in nature in that it tests the efficacy of FOP label types [10,11]. Such developmental/efficacy studies typically involve the comparison of several different FOP label formats or sub formats. The dependent variables are typically consumer assessments of product healthfulness or a similar intermediate response measure that is then related to the various FOP label types used in the research.

The distinction between efficacy and effectiveness research is subject to a degree of interpretation. It may be drawn on the basis of research conducted in “real world” (effectiveness) and controlled (efficacy) environments. The researchers chose to draw it on the basis of the dependent variable being used, with studies using the “final” dependent consumer variable of consumer choice being an “effectiveness” test and research using the “intermediate” consumer variables of attitude, understanding *etc.* as an ‘efficacy’ test. Both definitions have their limitations, and the only study that would satisfy both these reasonable criteria for “effectiveness” would be a very large scale, real world experiment under controlled conditions with consumer choice/purchase as a dependent variable. Such a research exercise is theoretically possible, but is beyond the means of all researchers, bar a state agency operating with total industry support and cooperation at all levels. No such studies have been conducted to date.

Those that do test the effectiveness of FOP labels by measuring their effect on consumer choice or intentions are predominantly based upon scanner data of actual sales, which can create issues of effective control over longitudinal datasets in a free retail environment [12,13,14,15].

The two very detailed reports by the US Institute of Medicine (IOM) [16,17] concluded that the evidence to support performance of reductive FOP labels was lacking, and that future efforts to influence dietary choice should be based upon evaluative formats. They made a series of specific recommendation that the FOP system used should be: (1) Standardized across all fresh and packaged foods in a supermarket; (2) Simple and easy to understand; (3) Ordinal; (4) Interpretative via use of colour and symbols; (5) Supported by an ongoing promotional campaign [17].

Lachat and Tseng made specific observations that the widespread introduction of FOP label systems was occurring before their effectiveness had been systematically tested, and they called for specific research in the area of direct FOP performance/effectiveness testing under controlled conditions [11]. In response to this call, an experimental exercise was undertaken in New Zealand to test the impact that TLL (hybrid) and PDI (reductive) FOP nutritional labels had on consumer choice behaviours towards breakfast cereals of high and low nutritional status when measured in the field [6]. A fully evaluative mark was not tested, as one was not available in New Zealand at the time.

The research results strongly indicated that consumers did “acquire” and process these labels, and that the labels consequently had a significant impact on consumer choice for all the cereal products that were tested. However, the impact on choice was not moderated by the information presented by the FOP labels. If an FOP label was present, then the consumers” selection intent rose significantly and consistently, regardless of whether the nutritional status being communicated to the consumer by the FOP label was high or low. This could be more properly described as a malfunction, rather than a failure of the FOP labels, as they were processed by the sample, but were not processed in the expected manner. However, as the sole purpose of an FOP label is to support consumer discrimination on the basis of nutritional status, these results indicate that both formats were entirely ineffective in the research situation.

The results of the New Zealand study suggested that the consumers tested were in fact (mis)using the reductive and hybrid labels as a fully evaluative “binary” cue, much like the “Swedish Keyhole” system or the term “organic”. As the vast majority of proposed and existing FOP nutritional label systems outside Scandinavia are untested in terms of their impact upon unprompted consumer choice behaviour, and are either reductive or hybrid types, [2,11,18,19,20,21] the researchers concluded that replication and development of this small initial testing exercise was urgently required.

**Figure 2:**
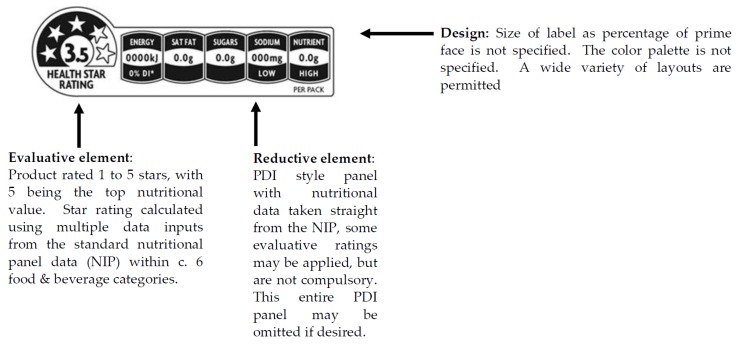
Health Star Rating (HSR) label.

At this point, in mid-2014, the Australian New Zealand Food Standards Authority (ANZFA) announced the introduction of a common FOP nutritional label system that would be voluntary throughout Australasia [22]. The “Health Star Rating” (HSR) FOP label is a hybrid type containing a reductive type PDI element, and a novel evaluative “star rating” system derived from the existing Energy Star Rating system for appliances [23]. It incorporates four of the five recommendations made by the IOM [16,17], lacking only heavy promotion. The style guide allows a degree of latitude as to how the label is presented, but the “star disk” is a consistent feature (Figure 2) [24]. The star ratings are calculated for a small number of broad food categories using the nutritional information provided elsewhere on the product using a website delivered calculator [25,26].

The HSR system was introduced without testing its impact on consumer choice. The “Star Disk” principle component of the HSR system is a hybrid type that lies well to the evaluative end of the reductive/evaluative continuum. It is similar to the format suggested by the second IOM report [17], and is well established as a system for indicating energy efficiencies in electrical goods in Europe, the United States and Australasia [17]. The format also bears considerable similarities to the proprietary “Guiding Stars” system owned by the US retailer “Hannafords”. The Guiding Stars system is one of the very few that has empirical evidence to suggest that it effectively supports consumer purchase discrimination at the point of sale [27].

However, there are also significant differences between the Hannafords and ANZFA system. Most notably, the Hannafords system is simpler (three stars *versus* five), proprietary, and was backed up by considerable targeted communication effort in a retail environment that was controlled by its owner when the research was conducted. It is also notable that both of these features of the “Guiding Stars” system were recommended by the second IOM report [17] (p. 4). The introduction of the HSR system was not without controversy, with some suggesting that the more established TLL system should have been deployed instead [28,29]. However, it should be noted that the TLL system is not as well established in Australasia as it is in Europe, has a very wide variety of presentation formats, with none of these dominant, and has, as yet, no consistent empirical evidence to support its effectiveness in influencing unprompted consumer choice [6].

It was clear therefore, that a much larger field test of this particular FOP label should be undertaken immediately, to establish if the new ANZFA HSR disk would influence consumer purchase behaviour as its proponents predicted, or if it would either not work at all or malfunction in the manner observed in the previously reported research [6]. Two research hypotheses were therefore developed for testing:

***H^1^***—The HSR FOP label would significantly influence consumer choice.
***H^2^***—The HSR FOP labels’ impact upon consumer choice would be moderated by variations in the 0.5–5 “star” rating expressed by the label.

## 2. Materials and Methods

### 2.1. Sample

**Figure 3:**
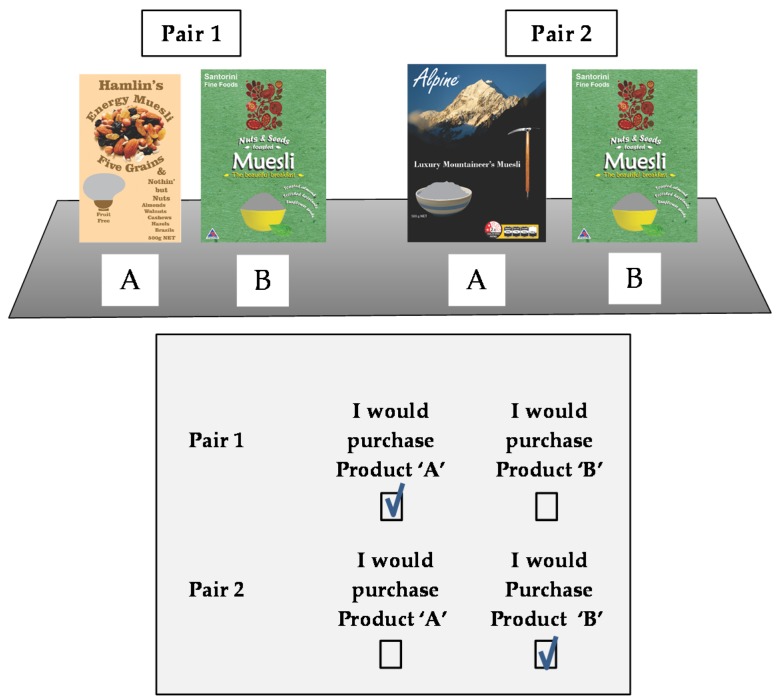
Choice task as presented to a consumer in Group 2 of the experimental sample.

The research was conducted over six weeks in October and November 2014, using a sample of 1200 consumers recruited by qualified intercept on exit from six major full service supermarket retail sites, located in the City of Dunedin, New Zealand, and its environs. All six stores were of the same format, and were owned by the same organization. The use of a comparator product (see Figure 3) meant that each consumer could evaluate two product pairs, yielding a total observation sample of 2400 (400 per site). This research received “category B” approval in 2014 from the Human Research Ethics Committee, University of Otago, New Zealand.

Intercepts were qualified by having purchased food items from the store on that visit, and by reporting that they had purchased breakfast cereals at least once in the previous month. A small incentive/courtesy of a novelty bar of chocolate was given for the short duration task, which generated a recruitment rate of c. 70% for all qualified exiting customers. 200 qualified consumers were recruited from each site in an exercise that took between three and five hours per site. All six exercises were run on a Friday starting at 10:00 a.m. 99.1% percent of those intercepted were regular store shoppers, with 71.7% being regular buyers of cereals. The average age of the qualified sample was 46 years, and 68.3% of the sample was female.

This recruitment approach did create a restricted sample. All instruments of parallel comparison, such as full factorials, fractional factorials and the Latin square rely upon the underlying homogeneity of their samples. This is especially so if replications within the sample are involved as they are in this case. Replications will increase the power of the experiment, but only if they are as homogenous, one with another, as possible. This does restrict the range of the experiment, and this trade-off in terms of external validity was made by the researchers in order to maximise the internal validity of the research.

### 2.2. Measurement

The dependent variable was discrete consumer choice, which is becoming an increasingly common and well recognized dependent variable in consumer behaviour research [30,31]. Individual consumers were presented with a pair of products, and were asked to indicate which one of them they would choose, with no allowance for an undecided or uncertain response. In this exercise each consumer was required to evaluate two pairs of products. A typical research environment for an individual consumer, with two pairs of products “A” and “B”, is shown in Figure 3, together with its associated response form.

The dependent variable used in the analysis was the percentage of the 100 consumers in each cell of the experimental design that chose product “A” (If 57 out of 100 chose “A”, then the input into the statistical analysis is 0.57 (57%). As long as over 30 choice events contribute to the average, then it can be treated as a parametric statistic in accordance with the Central Limit Theorum and used as an input to an analysis of variance.

### 2.3. Experimental Design

**Figure 4:**
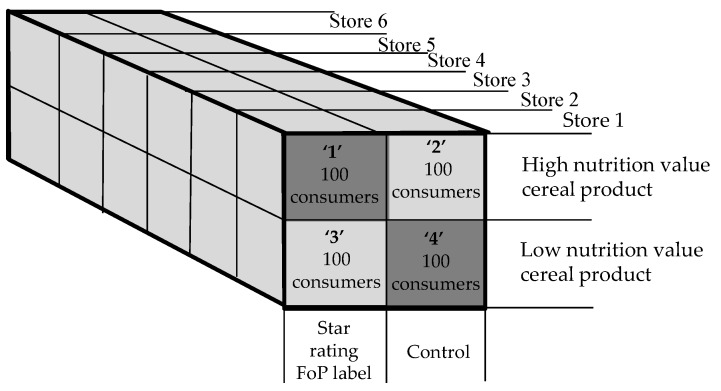
Experimental design.

A 2 × 2 factorial experiment with six replications was used, and the design is shown in Figure 4. In the 2 × 2 factorial design, there were two independent variables, FOP label and cereal product. Cold breakfast cereal was selected as the category for this study because: (1) It is a very heavily consumed category; (2) The packages are large and relatively consistent in their format; (3) The products within the category present a very wide nutritional status range, which made the presentation of high/low nutritional status treatments entirely plausible. A single category was used to achieve the highest possible level of internal validity. Each independent variable was at two levels: 1—present/2—absent for the FOP label, and 1—high/2—low nutritional status for the cold cereal product.

If the FOP star rating label was present (level 1), then it was placed on the prime facing of the product and was adjusted to exactly reflect the nutritional status of the product to which it was applied. As the HSR label style guide did not specify either color, size or a unique layout [21], the “full” layout version of the HSR (Figure 2) was set to 2.7% of the prime face of each product, and the required palette of the existing and very similar Australasian star energy rating for electrical appliances was used. The two levels of the cereal product were cold ready to eat muesli products; one with high (five star) nutritional status, (level 1) and one with low (two star) nutritional status (level 2). These products were emulations of two existing cold cereal products that were at the high and low extremes of nutritional status within this category respectively. The prices for the real products that the mock-ups emulated were used in the experiment and affixed, using the retailer’s standard “ticket”, to the platform upon which the products were presented. The analyses of all products used in the experiment is given in Table 1, together with their HSR ratings [25,26]. The TLL ratings for the United Kingdom are also given for the purposes of comparison [32].

**Figure 5:**
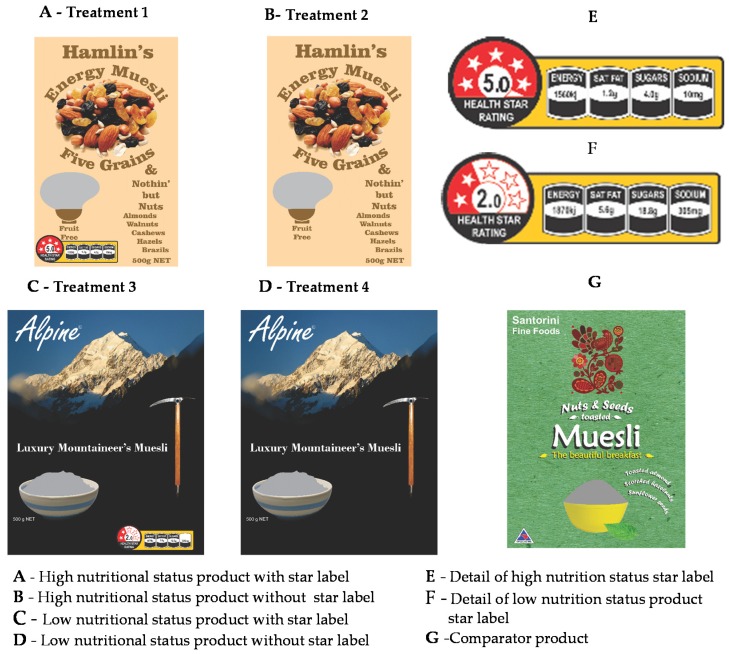
Front facings of the treatments used, comparator product and HSR FOP labels.

The four unique label/product treatment combinations were presented to the consumer sample as high quality complete product mock ups. The front facings of these four products are shown in Figure 5A–D. Details of the HSR labels applied to each product are shown in Figure 5E,F.

After recruitment, each consumer was randomly allocated by number to two equally sized groups. Group One evaluated treatments 1 and 4 (dark shaded in Figure 4), while Group Two evaluated treatments 2 and 3 (light shaded in Figure 4). This allowed each consumer to evaluate two products without becoming aware of the purpose of the research, by a process known as confounding in which several features of the product are changed simultaneously. This is best understood by looking at the consumer task shown in Figure 3. While the FOP label was present on one product and absent on the other, the two products upon which the FOP label was presented were different, thus denying the consumer any basis upon which they could deduce that the FOP symbol was the variable of interest. If the purpose of the research can be concealed from the experimental population, then their choice behaviour can be treated as a *revealed* rather than *reported* behaviour, and thus any chance of reporting and associate bias can be discounted [33].

The four choice tasks used in the experiment were created by placing each of the four treatment combinations next to a common comparator product (Figure 4, G). The consumer was asked to indicate which of the two products they would choose by ticking either the “A” (treatment combination) or “B” (comparator) boxes on their response form. Each treatment/comparator combination was evaluated by 100 consumers. Order effects were suppressed by the treatments being reversed every tenth consumer.

### 2.4. Analysis

After coding, the data were analyzed in Excel using a simple two way analysis of variance, which provided an estimate of the significance of: (1) The main effects of the FOP label treatments (present/absent); (2) The nutritional status of the two products (high/low) and; (3) The product *x* FOP label interaction between the presence/absence of the FOP label and the nutritional status of the product. The nature of these main effects and interactions were then further analyzed by plotting the four points generated by the data on a chart. The six sites at which consumers were recruited were treated as replications rather than as an independent variable in this analysis, as measures were taken to ensure that the sites, recruitment times and consumer samples were as equivalent as possible.

## 3. Results

**Figure 6:**
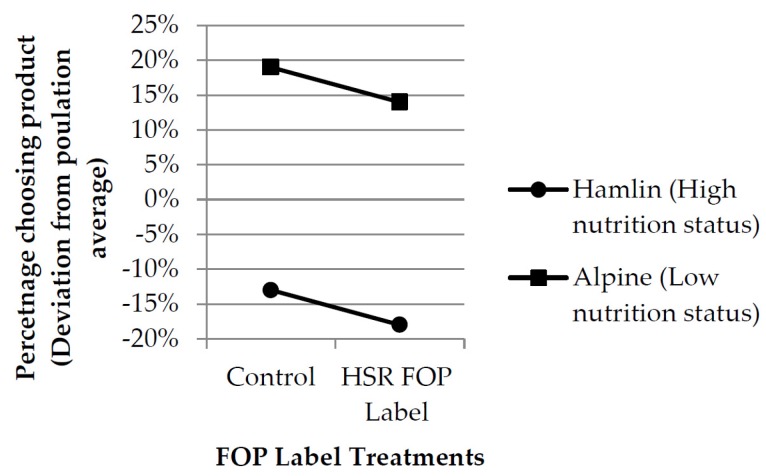
Graphical presentation of results.

The results of this research are summarized in Table 2 and Figure 6. Significant and substantial main effects were observed for both product and FOP treatments. The “Alpine” muesli was favored strongly over the “Hamlin” muesli, despite the latter’s much higher nutritional status. In both cases the presence of the HSR FOP significantly depressed purchase intention, although this effect was minor compared to the main effect of the products themselves.

The level of product *x* FOP label interaction reported by the analysis of variance was not merely insignificant, it was non-existent. The same comment can be made for the levels of experimental error. Error levels that are this low are quite unusual in the social sciences. However, this particular form of classical research design, when executed in the field with care, is routinely capable of generating very low errors of this type when consumer samples are as large and homogenous as they are here [34,35].

The use of a replicated full factorial design, with a very low and insignificant level of interaction reported in the results, means that it can be stated with a high degree of confidence that the response to the labels and the response to the products were independent of one another, and thus the high degree of separation observed in the product evaluations did not influence the HSR FOP label evaluations.

## 4. Discussion

The objective of this research was to test the impact of the HSR label on consumer choice with as simple and direct a methodology as possible. The ANZFA literature that supports the program indicates that there is no specific target market for the label [25]. The researchers therefore selected a homogenous but generic sample of retail cereal shoppers’, acquired by qualified exit intercept from New Zealand’s leading retail store chain on a specific day of the week. The sample amounts to some 1.2% of the entire population of a cosmopolitan university city.

In order to maximize the internal validity of the exercise, a very tight research focus was adopted, with two very specific hypotheses and a very tightly controlled and focused research environment that nevertheless relied upon a field rather than a laboratory environment. The use of a homogenous sample, single product type, a full factorial design, a revealed consumer response and the choice dependent variable minimized the assumptions made to the greatest extent that was possible.

***H^1^:*** “*The HSR FOP label would significantly influence consumer choice*”. is supported by this result as the consumer samples clearly took significant notice of the HSR FOP labels, and their presence exerted a significant influence upon consumer choice behaviour with regard to both products. The presence of the HSR FOP nutritional label consistently reduced consumer preference. This is shown by the strong downward slope of the two lines in Figure 6. If the labels had failed to impact upon choice, then the two lines in Figure 6 would be horizontal. This result is consistent with the earlier research undertaken by these authors [6] in that the TLL and PDI labels also had a significant main effect impact upon consumer choice. However, the earlier research results indicated that the PDI and TLL FOP cues consistently increased consumer preference, whereas in this research, the HSR consistently reduced it.

The reasons for these differences in main effects are at present completely unknown, but may have their origins in subconscious consumer processing of the very different graphic designs of the two label systems. The mechanics of consumer reactions to package graphic design is at present poorly understood, but graphic design has been shown to have considerable and unpredictable effects on food consumer preference behaviour [35].

***H^2^****:* “*The HSR FOP labels’ impact upon consumer choice would be moderated by variations in the 0.5–5 ‘star’ rating expressed by the label*”. is not supported by this result. The nutritional status reported by the two HSR FOP labels used varied widely between the two products, but this variation in status reported by the FOP had no observable differential impact upon consumer product choice. The two lines in the chart in Figure 6 reflect this observation as they are almost precisely parallel with one another. This is also supported by the almost non-existent level of product *x* FOP label interaction reported in the analysis of variance, despite the very substantial main effects observed for both product and label in the same table. Had the nutritional status expressed by the HSR FOP label been a significant input to the consumer choice, then this effect would have been expressed as a product *x* FOP label interaction in the analysis of variance table, and the two lines on the chart would be at a distinct angle relative to each other.

**Figure 7:**
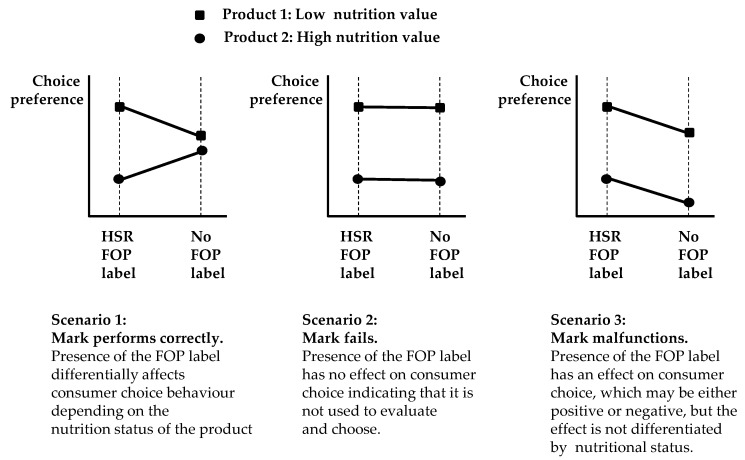
Plots of results related to differing outcomes.

Had the labels failed outright, then they would not have been used in the consumer evaluation and choice process. As a consequence there would have been no observable main effect for FOP label, and the two lines shown in the chart in Figure 6 would have been parallel and horizontal. These three possible outcome scenarios are reproduced graphically in Figure 7.

The results achieved here are exactly consistent with scenario three of Figure 7. They indicate that this large consumer sample’s choice behaviour was significantly depressed with regard to products that presented the HSR FOP, and that the consumer sample was also either unwilling, or unable, to use its informational features as an input to a differential evaluation of a food product in the manner expected by ANZFA. This outcome indicates a significant functional failure of the HSR FOP label in this research environment.

If this result is an outcome of a fundamental mismatch between the HSR FOP’s structured cognitive input, and a dominant, unstructured non-cognitive consumer decision process at the retail point of sale, then it is may be a highly intractable issue—if it is tractable at all. The fact that ***H^1^*** is supported, while ***H^2^*** is not, does suggest that this may be a possibility.

**Figure 8:**
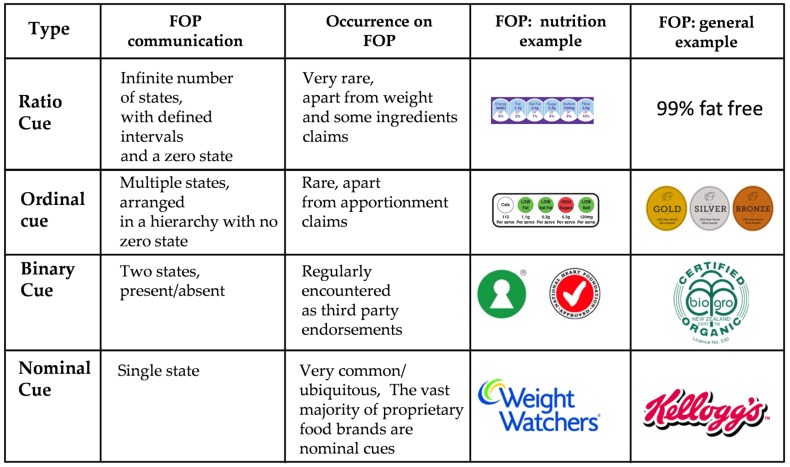
Nominal, binary, ordinal and ratio cues.

The concept of potential failure via mismatch may be more easily understood when the nature of consumer communication via the package at the point of sale is considered. Figure 8 shows a typology of cues, and their relative frequency of occurrence in the retail food point of sale. Ninety nine percent plus of all cues presented to the consumer on the package at the point of sale are nominal (brands) or, much more rarely, binary (third party endorsements). Both of the types are processed by the consumer on the basis of their presence or absence, not by any range of states that they express—however simple.

Ordinal and ratio cues are virtually unknown as on-package consumer communication elements beyond the various FOP label formats that are available around the World. “The Gold” “Silver” and “Bronze” wine awards are one of the very few deployed examples of an ordinal cue that are known [36]. Nominal/binary cues and their related consumer processing pathways are thus the norm in retail food communication, a situation that is supported by commercial brand consumer research and communication budgets that run into the tens of billions of dollars worldwide [37,38]. The scale upon which nominal/binary cue based communication occurs at the retail point of sale means that any cue format that does not conform to this type, such as an ordinal cue, is likely to face a very challenging environment.

Even when presented by the one truly common ratio cue, price, research has shown that consumers will “convert” this information to a nominal/binary form if they are given the opportunity to do so (e.g., is it “on special” or not) [39]. Retailers are well aware of this consumer tendency and exploit it widely via price/special branding programs that facilitate and encourage this process of ratio to binary cue conversion by the consumer [40,41].

It is now possible to relate this argument to the plot shown in Figure 6, and to Scenario 3 of Figure 8. Food consumers have been trained to react subconsciously to nominal or binary cues, presented as pictorial icons. Commercial food brands are the dominant type of such icons. As the presentation of these individual iconic cues is highly consistent, the individual consumer’s subconscious reaction to them is too. The massive intellectual property valuations attached to commercial brands and their registered designs is based upon the assumption that if these brand icons are applied to an appropriate food product, then, ceteris paribus, the plot of the type shown in Scenario 3 of Figure 8 will occur [42].

Figure 6 indicates that the HSR label was being processed by this sample in the same way that they would process an iconic brand. The results show that it was acquired and processed by the sample, and it was having a significant impact on their choices, but this impact was not moderated in any way by the number of stars presented on the label. The label was being treated as a single integrated iconic cue by the sample. The fact that the main impact of the HSR label was to significantly depress consumer selection is interesting, but not directly relevant to the HSR label’s failure to create a significant consumer distinction based upon the number of stars presented. Any attempt to explain this consistent depression in preference would be purely speculative at this point.

## 5. Conclusions

The result generated in this research is that the HSR FOP nutritional label is processed in some way by consumers in the sample, but it does not support consumer discrimination on the basis of the nutritional status reported by it. This is consistent with the consumer sample internally “converting” the ordinal HSR nutritional label into a nominal/binary type cue, and then using it in this form as an input for the purposes of supporting decision processing in a manner that is more familiar to them. This conversion process is both theoretically plausible, and has been observed.

These results suggest a significant functional failure of the HSR FOP system in this instance. The implications of this are not encouraging for the backers of the HSR FOP, or of any of the other major hybrid/ordinal FOP systems. More field trials to test the relative impact of reductive, hybrid and evaluative FOP systems upon unprompted, revealed consumer choice are required to either confirm, modify, or dismiss this result.

While the researchers are satisfied that this research has a high degree of internal validity, there are some very clear limitations which relate to its very tight focus and consumer sample. The results were gathered within a single city in New Zealand, and any extrapolation to the entire population of New Zealand and Australia would have to be undertaken with caution. The same comments apply to the products used, which were restricted to two products in the cold cereals category. The area of consumer response to FOP labels represents an environment of considerable complexity, and considerable further situation-specific FOP label research needs to be conducted before generic statements such as “Food labelling would increase the amount of people selecting a healthier food product by about 17.95% (confidence interval: by about 17.95% (confidence interval: +11.24% to 22.46%)” [43] (p. 201) can be made with any degree of confidence or credibility.

The hypothetical choice/purchase situation also has the capacity to introduce several forms of bias including; social desirability bias relating to the consumer’s perception that this is an observed behaviour, forced exposure to a far more limited choice set than is usually available, and lack of tangible consequences for a decision that does not involve an actual purchase.

The HSR third-party FOP mark itself is of a type that is unique to Australasia, and other major markets are pursuing other FOP designs such as the TLL and PDI format. While the TLL and PDI are of the same highly atypical ordinal/ratio cue type as the HSR label, and are in fact both slightly more extreme in this regard, a similar performance outcome for these label systems cannot be assumed on the basis of this commonality.

## Figures and Tables

**Table 1:** Nutritional status of the three products used via nutrition information panel (NIP), and related HSR and traffic light label (TLL) front of pack FOP ratings.

Nutrient	Unit	Per 50 g Serving			Per 100 g			HSR FOP Rating (0.5 to 5 Stars)			TLL FOP Rating (Grn/Amb/Red)		
		Alp	Ham	San	Alp	Ham	San	Alp	Ham	San	Alp	Ham	San
**Energy**	(kj)	935	780	950	1870	1560	1560						
	(Cal)	223	186	227	446	372	454						
**Protein**	(g)	3.0	6.1	6.5	6.0	12.2	13.0						
**Fat, Total**	(g)	8.8	4.2	8.8	17.6	8.4	17.7				Amb	Amb	Amb
**Sat. Fat**	(g)	2.8	0.6	1.3	5.6	1.2	2.6				Red	Grn	Amb
**Carb’te, Total Sugars**	(g)	29.6	28	28.2	59.1	56	56.4						
	(g)	9.4	2.0	7.6	18.8	4.0	15.2				Red	Grn	Red
**Dietary Fibre**	(g)	2.7	5.3	3.8	5.3	10.6	7.6						
**Sodium**	(mg)	153	5	38	305	10	74				Amb	Grn	Grn
**Potassium**	(mg)	173	7	45	345	14	90						
**Overall Health Star rating (0.5–5 stars)**	star							2 stars	3 stars	5 stars			

Alp = Alpine low nutrition status product; Ham = Hamlin high nutritional status product; San = Santorini’ comparator product. Grn= green; Amb = Amber; Red = red.

**Table 2:** Analysis of variance table.

Source of Variance	Sum of Squares	Degrees of Freedom	Mean Square	F Ratio	Significance
Total	0.6770	23			
Products	0.6107	1	0.61	226.4	*** *p* = 0.01
HSR FOP label	0.0123	1	0.01	4.6	** *p* = 0.05
Package *x* FOP label interaction	0.0000	1	0.00	0.0	NS
Error	0.0539	20	0.00		

## Abbreviations

The following abbreviations are used in this manuscript:

FOP	Front of pack
NIP	Nutrition information panel
FMCG	Fast moving consumer goods
PDI	Percentage daily intake
TLL	Traffic light label
RDI	Recommended daily intake
TLS	Traffic light system
ANZFA	Australia/New Zealand Food Standards Authority
HSR	Health Star Rating
